# Protrusion of a loop‐shaped 0.035‐inch wire without the fracture of the self‐expanding nitinol stent: A case report and experimental study

**DOI:** 10.1002/ccr3.1988

**Published:** 2019-01-13

**Authors:** Eiji Karashima, Daizo Kawasaki, Shunsuke Yoda, Shioto Yasuda, Takeo Kaneko

**Affiliations:** ^1^ Department of Internal Medicine, Division of Cardiology Shimonoseki City Hospital Yamaguchi Japan; ^2^ Cardiovascular Division, Department of Internal Medicine Morinomiya Hospital Osaka Japan

**Keywords:** endovascular treatment, in‐stent restenosis, knuckle wire technique, peripheral artery disease, superficial femoral artery

## Abstract

We report a case of a loop‐shaped 0.035‐inch wire protruding through self‐expanding nitinol stent struts. Our in vitro experiment suggests that, even if there are no stent fractures, the loop‐shaped 0.035‐inch wire has a potential to protrude through the struts of the self‐expanding nitinol stents.

## 1 INTRODUCTION

Endovascular therapy (EVT) is an established treatment strategy for peripheral artery disease (PAD).^1^ In particular, deployment of a self‐expanding bare nitinol stent is a standard procedure for femoropopliteal (FP) artery stenosis and occlusion, showing improved primary patency and clinical outcomes in patients with PAD.^2^ Despite the favorable patency rate of the nitinol stent, the growing number of FP stenting cases is increasing the incidence of in‐stent restenosis (ISR).^3^ In‐stent restenosis chronic total occlusion (ISR‐CTO) is one of the most challenging lesions to treat, and the success rate varies depending on operator’s expertise, lesion complexity, and interventional therapeutic strategy. The knuckle wire technique is an excellent therapeutic option for ISR‐CTO revascularization because it does not require a dedicated device to cross the occluded stent. Moreover, this technique is safely guided by the roadmap obtained with the prior stent implantation under fluoroscopic image monitoring of the wire position.^4^ However, in some cases, a thick (eg, 0.035‐inch) wire protrudes through the slit of the stent strut in a loop‐shaped manner, and the mechanisms for the wire loop formation outside the nitinol stent framework are not well understood.

Therefore, we report a case of PAD presenting with ISR‐CTO and complicated by a loop‐shaped 0.035‐inch wire protrusion, along with the results of our experimental study.

This study was performed according to the principles of the Declaration of Helsinki, and written informed consent was obtained from the patient.

## 2 CASE PRESENTATION

**Figure 1:**
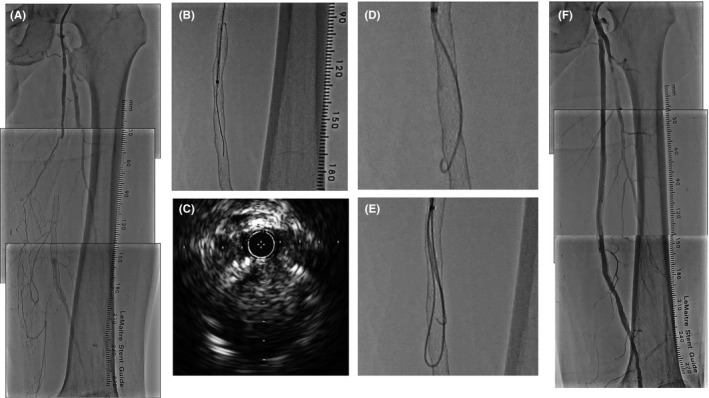
Angiographic images and an intravascular ultrasound (IVUS) image. Control angiography revealed total superficial femoral artery occlusion (A). The GOGO catheter was advanced into the nitinol stent, which was confirmed by IVUS (B). IVUS finding when the IVUS protruded through the stent struts (C). Angiographic findings of the 0.035‐inch small J‐type hydrophilic guidewire (D) and 0.035‐inch loop‐shaped wire (E) protrusion. Angiographic image at the end of the procedure (F)

A 75‐year‐old man in whom a 6.0 × 60‐mm self‐expanding bare nitinol stent (Misago; Terumo, Tokyo, Japan) had been deployed to the stenotic midportion of the left superficial femoral artery (SFA) 9 months prior was admitted to our hospital with a recurrence of intermittent claudication on the left side. He had hypertension, dyslipidemia, diabetes mellitus, and a history of coronary artery bypass grafting. The ankle‐brachial index was 0.81 on the right and 0.45 on the left, and contrast computed tomography and angiography revealed occlusion of the left SFA. The beginning of the occlusion was about 5 cm proximal to the stent, and its end was on the distal side of the stent (Figure 1A). A 6‐F straight guiding catheter (Parent plus; Medikit, Tokyo, Japan) was placed proximal to the CTO entrance stump. Intravascular ultrasound (IVUS)‐guided wiring was performed with 0.014‐inch wires (Chevalier Tapered 15; Cordis Corporation, Miami Lakes, FL, USA, and Jupiter Tapered 45; Boston Scientific, Marlborough, MA, USA) to cross over the plaque located within a few centimeters from the CTO entrance.^5^ IVUS (Eagle Eye Platinum ST Catheter; Philips Corporation, San Diego, CA, USA) could approach the proximal edge of the Misago stent, but could not be advanced into the internal parts of the stent. Balloon angioplasty with a 3.0 × 20‐mm balloon catheter (Shiden; Kaneka Medix Corporation, Tokyo, Japan) was performed because a few centimeters of the 0.014‐inch wire were located within the stent, which was confirmed by rotation angiography. The GOGO catheter (Medikit) was advanced antegradely to the point where it was confirmed to be within the stent by IVUS (Figure 1B). The 0.014‐inch wires were protruded through the stent struts. The IVUS‐guided technique was attempted, but the IVUS also protruded through the stent strut (Figure 1C). The 0.014‐inch wire and the IVUS were then removed from the GOGO catheter, and a knuckle wire technique was performed with a 0.035‐inch small J‐type hydrophilic guidewire (Radifocus; Terumo). The small J‐type and loop‐shaped 0.035‐inch wire was also protruded through the stent strut (Figure 1D,E). We terminated the wire crossing by the knuckle wire technique with the 0.035‐inch wire. An IVUS‐guided tapered 0.018‐inch wire (Astato; Asahi Intecc Corporation, Aichi, Japan) could cross into the occluded stent lumen. Subsequent to a successful balloon angioplasty to the occluded stent, an 8.0 × 150‐mm bare nitinol stent (S.M.A.R.T. control; Cordis Corporation) implantation was performed to the proximal side of the Misago stent. We were able to treat this ISR‐CTO case without any complications.

## 3 EXPERIMENT

In our case of ISR‐CTO complicated by procedural wire protrusion, we investigated the mechanism of the loop‐shaped protrusion of the 0.035‐inch wire using in vitro stent occlusion models with three types of stents. We prepared the self‐expanding nitinol stents, including S.M.A.R.T. control (Cordis Corporation), INNOVA (Boston Scientific), and Misago (Terumo), with a diameter of 6 mm. One side of the self‐expanding stents was occluded by the same‐sized balloon (Sterling 6.0 × 40 mm; Boston Scientific) inflated by nominal pressure, and a 0.035‐inch wire was inserted from the other side.

**Figure 2:**
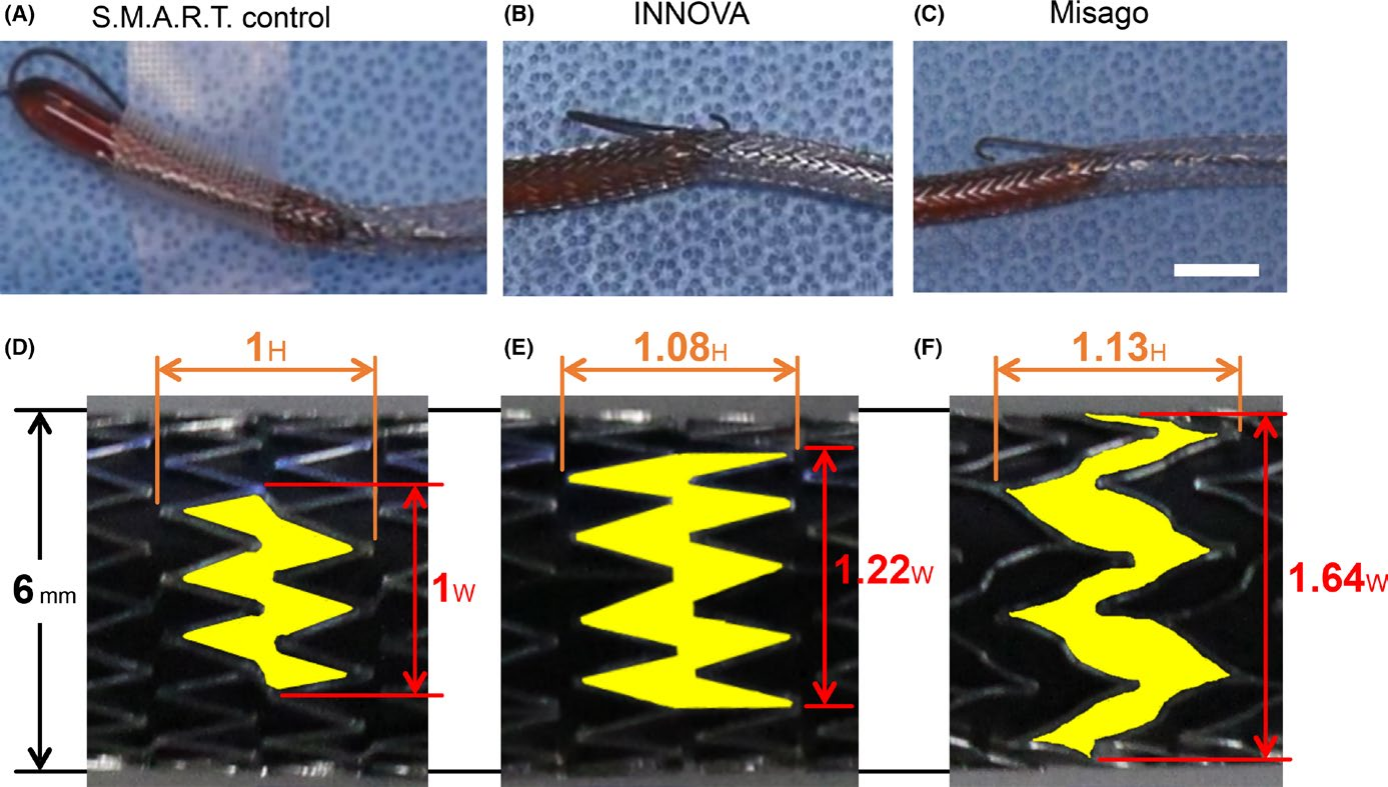
Pictures of the 0.035‐inch small J‐type hydrophilic guidewire protrusion from the S.M.A.R.T. control (A), INNOVA (B), and Misago (C) stents, and magnified pictures of the S.M.A.R.T. control (D), INNOVA (E), and Misago (F) stents. These stents have the same diameter of 6 mm (black line). To clarify the difference in the cell design of each stent, the cells are marked in yellow. The white bar indicates 10 mm for (A), (B), and (C). Having defined the height and width of a cell of S.M.A.R.T. control as 1 H and 1 W, respectively, as shown in (D), those of the INNOVA and Misago were found to be 1.08 H and 1.22 W, and 1.13 H and 1.64 W, respectively

To make a protrusion from the S.M.A.R.T. control stent, we advanced the 0.035‐inch small J‐type hydrophilic guidewire manually. The wire was bent inside the stent, and the loop‐shaped wire was slipped into the space between the dilated balloon and S.M.A.R.T. control stent. During the experiment, the loop‐shaped 0.035‐inch wire did not protrude from the S.M.A.R.T. control stent (Figure 2A). In contrast, the loop‐shaped 0.035‐inch wire protruded from the INNOVA and Misago stents. To make a protrusion from the INNOVA stent, we pushed the 0.035‐inch small J‐type hydrophilic guidewire strongly. When the wire protruded from the INNOVA stent, the wire was bent (Figure 2B). To make a wire protrusion from the Misago stent, we did not have to push the 0.035‐inch small J‐type hydrophilic guidewire so strongly. The shape of the wire was not changed when it protruded from the Misago stent (Figure 2C).

## 4 DISCUSSION

We report a case of PAD with ISR‐CTO that was successfully treated; however, the knuckle wire technique failed. A loop‐shaped 0.035‐inch wire does not usually protrude through stent struts. We speculate that the possible cause of this rare phenomenon was that the stent strut had enough space to allow protrusion of the loop‐shaped wire. To assess this possibility, we checked whether a 0.035‐inch wire could pass through the slit of stent struts in the loop configuration.

Based on our experiment, the loop‐shaped 0.035‐inch wire protrusion was dependent on individual nitinol stents (Figure 1). To determine the cause, we compared the cells of the three types of stents, which are fabricated by lasercutting of a nitinol tube. To obtain balanced flexibility and radial force tolerance, each stent has a different cell design. The width of the cells of each stent is also different (Figure 2). The S.M.A.R.T. control stent has the narrowest cell, while the Misago stent has the widest cell. The cell width of Misago is almost half around the stent. We expected that when we pushed the stiff ISR‐CTO with a loop‐shaped 0.035‐inch wire, part of the stent cells must have been stretched and made enough space for the wire to protrude. The width of the stent cells should determine the different ways of protrusion of the loop‐shaped 0.035‐inch wire from each stent.

In our case, the possibility of stent fracture during the EVT procedure could not be excluded. However, we could not find any reports investigating the loop‐shaped 0.035‐inch wire protrusion. The results of our experiment suggest that the probability of the loop‐shaped 0.035‐inch wire protrusion depends on the width of the stent cells and not always on stent fracture.

The experimental study had several limitations. The occlusion was made by a balloon in our experiment, but vascular lesions are not homogenous because of the presence of various substances, such as plaques, thrombi, and calcified matrices. In addition, the pushing force of the wires was not measured because the wire protrusions were obtained manually. Further investigation is needed to apply our experimental results to clinical practice.

In conclusion, we report a rare case of a loop‐shaped 0.035‐inch wire protrusion through self‐expanding nitinol stent struts during the knuckle wire technique for the femoropopliteal ISR‐CTO. In addition, the results of our experiment suggest that the loop‐shaped 0.035‐inch wire protrusion may occur without stent fracture. In the case of a loop‐shaped wire protrusion, terminating the knuckle wire technique and changing the method should be useful to cross the wire into the ISR‐CTO.

## CONFLICT OF INTEREST

None declared.

## AUTHOR CONTRIBUTION

EK: gathered the patient and experimental data, performed a literature review, and wrote the manuscript. DK and SY: provided clinical data. SY and TK: were involved in overall supervision of the paper. All authors read and approved the final manuscript.
